# Japanese pediatric patient with moderately active ulcerative colitis successfully treated with ustekinumab: A case report

**DOI:** 10.1097/MD.0000000000028873

**Published:** 2022-02-18

**Authors:** Toshihiko Kakiuchi, Masato Yoshiura

**Affiliations:** Department of Pediatrics, Faculty of Medicine, Saga University, Saga, Japan.

**Keywords:** child, Japanese, ulcerative colitis, ustekinumab

## Abstract

**Rationale:**

Ustekinumab is effective in the treatment of adult Crohn disease (CD) and ulcerative colitis (UC). However, data on its efficacy and safety in pediatric CD and UC are limited. To the best of our knowledge, there are no reports of Japanese children with UC treated with ustekinumab in the long-term.

**Patient concerns:**

A 14-year-old man with diarrhea and bloody stools was referred to our hospital. Colonoscopy revealed total colitis-type UC. His pediatric UC activity index score was 50, indicating moderately active UC.

**Diagnoses:**

Ulcerative colitis.

**Interventions:**

Infliximab was introduced because of steroid-resistant refractory UC; however, secondary ineffectiveness was observed 17 months later. Therefore, ustekinumab was administered along with prednisolone (16 years of age).

**Outcomes:**

The patient achieved UC remission after ustekinumab treatment, leading to maintained remission without side effects.

**Lessons:**

To the best of our knowledge, this is the first pediatric case of moderately active UC successfully treated with ustekinumab in Japan. Ustekinumab combined with steroids is an effective and safe induction therapy for UC.

## 1 Introduction

Ustekinumab is a first-in-class therapeutic human immunoglobulin-G1 kappa monoclonal antibody that binds to interleukin-12 and interleukin-23, which are cytokines that modulate the function of lymphocytes such as T-helper (Th)-1 and Th17 cell subsets.^[1]^ Ustekinumab is effective in treating adult Crohn disease (CD) and ulcerative colitis (UC).^[^2^–^6^]^ However, data on its efficacy and safety in pediatric CD and UC are limited,^[7]^ although its effectiveness has recently been reported to be similar to that in adult CD and UC.^[^8^–^10^]^ The induction dose for pediatric UC is a weight-based intravenous loading dose followed by subcutaneous injections of 90 mg ustekinumab for maintenance every 8 to 12 weeks^[9]^; this dosing is the same as that for adult UC.

In March 2017, ustekinumab was approved in Japan as a new treatment drug for moderately to severely active CD in adults and was permitted in March 2020 for use in adult UC. Presently, we found no reports of Japanese children with UC treated with ustekinumab in the long-term. Herein, we report a Japanese pediatric case of moderately active UC that was successfully treated with ustekinumab.

## 2 Case report

**Figure 1:**
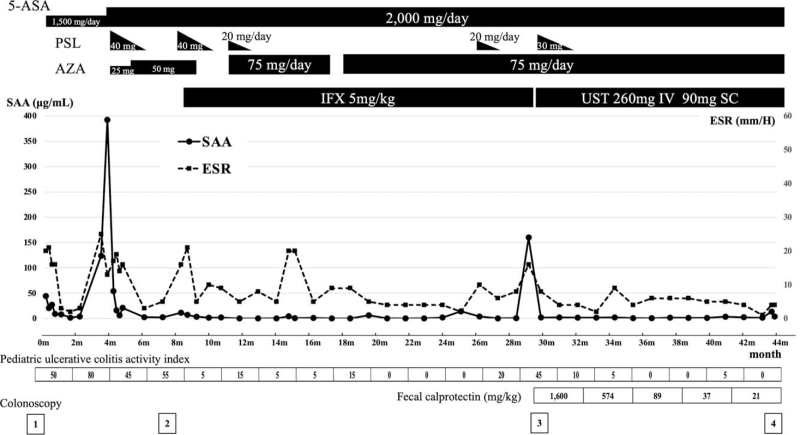
Patient's clinical course and treatment for ulcerative colitis. Although he was treated with 5-aminosalicylate acid, prednisolone (PSL), and azathioprine, remission could not be maintained. With a diagnosis of steroid-dependent ulcerative colitis, infliximab (IFX) therapy was started. Although remission was maintained for 17 months, IFX demonstrated secondary ineffectiveness; thus, ustekinumab combined with PSL was introduced. Re-remission was rapidly induced, and remission was maintained without side effects thereafter. 5-ASA = 5-aminosalicylate acid; AZA = azathioprine; ESR = erythrocyte sedimentation rate; IFX = infliximab; PSL = prednisolone; SAA = serum amyloid A; SC = subcutaneous injection; UST = ustekinumab.

A 14-year-old boy was referred to our hospital with chief complaints of diarrhea and bloody stools that had lasted for 4 months. Immediately after the passage of bloody stool, the attending physician of the former clinic performed total colonoscopy (TCS) under sedation. TCS showed diffuse reddish and edematous mucous membranes that bled easily and edema, along with multiple small erosions from the cecum to the rectum (Fig. 1). Pathological findings of the biopsy specimens from the sigmoid colon revealed acute and chronic inflammatory cell infiltration, crypt abscesses, and goblet cell depletion. No pathological bacteria were detected in the stool cultures. Hence, the patient was diagnosed with total colitis-type UC (Mayo endoscopic score 2). At that time, his pediatric ulcerative colitis activity index (PUCAI) score was 50 and serum inflammatory biomarkers were elevated (serum amyloid A, 48 g/dL; erythrocyte sedimentation rate, 142 mm/h).

**Figure 2:**
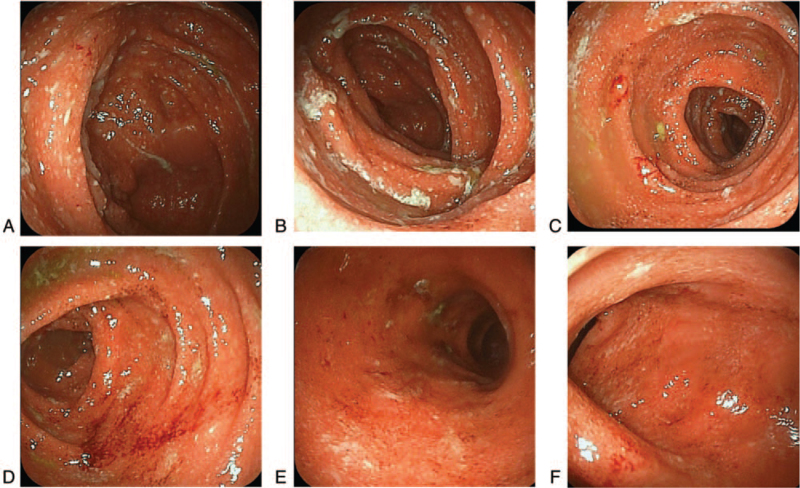
Endoscopic images during diagnosis. The mucous membrane was reddish, and it bled easily. Edema was also observed along with multiple small erosions from the cecum to the rectum (A: cecum, B: ascending colon, C: transverse colon, D: descending colon, E: sigmoid colon, F: rectum) (Mayo endoscopic score [MES] 2).

As shown in Fig. 2, the patient was initially treated with 5-aminosalicylate acid (5-ASA), which improved his symptoms. However, his gastrointestinal symptoms worsened after 3 months; therefore, he was referred to our hospital. Considering the relapse of UC, his 5-ASA dose was increased, and he received 1 mg/kg prednisolone (PSL) and azathioprine (AZA); consequently, his symptoms stabilized again. Two months after the end of PSL tapering, his abdominal symptoms recurred; thus, resuming PSL only slightly improved his symptoms. Finally, the patient was diagnosed with steroid-resistant refractory UC (PUCAI score, 55). According to the guidelines for pediatric UC, infliximab (IFX) was introduced as the first biologic.^[^11^,^^12^^]^ As a result, clinical remission was promptly achieved (PUCAI score 5). After 6 weeks, diarrhea recurred (PUCAI score: 15); nonetheless, the symptoms subsided with PSL (20 mg/day) and AZA resumption. Subsequently, the patient achieved clinical remission over 15 months.

**Figure 3:**
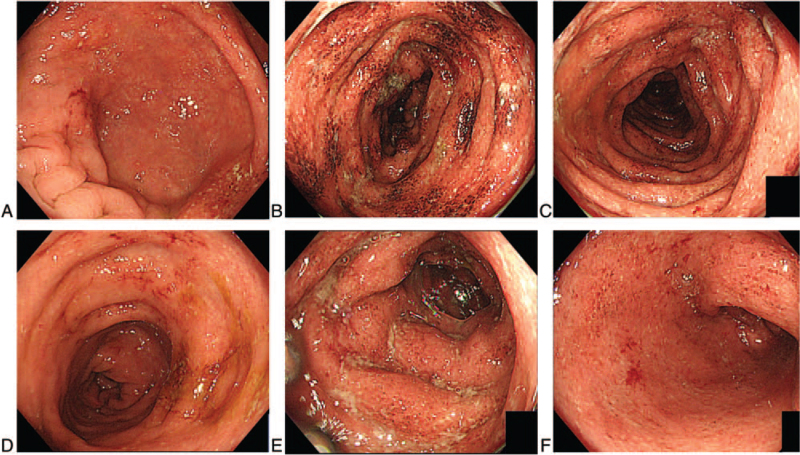
Endoscopic images immediately before ustekinumab administration. From the cecum to the rectum, disappearance of the blood vessel fluoroscopic image, adhesion of white moss, and formation of aphthae were observed all around (A: cecum, B: ascending colon, C: transverse colon, D: descending colon, E: sigmoid colon, F: rectum) (MES 2). MES = Mayo endoscopic score.

After 17 months of IFX, his abdominal symptoms (diarrhea and bloody stools) worsened. Despite additional treatment with PSL (20 mg/day), his symptoms did not improve. TCS was performed again to determine UC deterioration (Mayo endoscopic score 2) (Fig. 3). These findings indicated UC relapse (PUCAI score, 45). The trough level of IFX was below the cut-off value, and the anti-IFX antibody was positive; thus, IFX demonstrated secondary ineffectiveness. Hence, we selected ustekinumab as the second biologic; at this point, the patient was 16 years of age. He received 260 mg of ustekinumab intravenously on day 1 and 90 mg subcutaneously every 8 weeks thereafter.^[13]^ After 8 weeks, his abdominal symptoms improved quickly, reaching clinical remission at 16 weeks (PUCAI score: 5). Continuous administration of ustekinumab every 8 weeks maintained clinical remission, and 60 weeks after ustekinumab introduction, TCS revealed endoscopic remission. No side effects were observed during the course of ustekinumab administration.

## 3 Discussion

The clinical course of this case provided 2 important indications. First, ustekinumab was safe and effective against moderately active UC with anti-tumor necrosis factor (TNF) failure in the present case.

Ustekinumab is effective and safe for inducing and maintaining remission in adult patients with moderate to severe UC.^[^3^,^^5^^,^^6^^]^ Although the off-label use of ustekinumab in the pediatric population is increasing, data on its use in children remain scarce, especially for UC.^[14]^ Only 3 observational cohort studies on CD refractory to anti-TNFα therapies have reported the efficacy and safety of ustekinumab in children.^[^8^,^^9^^]^ Recently, Dayan et al^[9]^ conducted an observational study including 52 children and young adults (38 children, 81% with CD) and highlighted a higher efficacy among biologic-naive patients than among biologic-exposed patients (90% vs 50%; *P* = .03). A separate retrospective cohort of 44 children demonstrated similar long-term outcomes at 12 months.^[8]^ Thus, ustekinumab may be effective in children with inflammatory bowel disease (including a small proportion of UC). However, larger cohort studies are needed to validate the efficacy, safety profile, and optimized dosing regimen in children. This is the first reported pediatric case of moderately active UC that was successfully treated with ustekinumab in Japan. UC morbidity has racial differences,^[15]^ and one of the reasons may be the effect of UC-related genes and human leukocyte antigens. In a large-scale genome-wide association study from Europe and North America, approximately 50 UC-related gene regions were identified, but the results were different from those from Japan.^[^16^,^^17^^]^ Similarly, the effects and safety of therapeutic agents, including biologics, on UC may vary by race. A small retrospective study involving adult East Asian patients with UC (Japanese, 107; Korean, 26) reported the efficacy and side effects of ustekinumab for UC.^[18]^ There was no significant difference in the frequency of adverse effects between ustekinumab and the placebo. However, data on Japanese children remain scarce; therefore, accumulation of such data is needed.

Second, the introduction of ustekinumab with temporary administration of PSL resulted in quicker remission than that without PSL. The present case involved anti-TNF failure; thus, we expected that the effect of ustekinumab would take time to appear. Therefore, steroids were administered again to improve the patient's quality of life, considering the side effects of the increased risk of infection. The response to ustekinumab in CD usually appears in weeks.^[19]^ The extent to which ustekinumab can be used for UC, which may progress faster than CD, remains controversial. In a patient with more “severe” disease, using IFX or tofacitinib may be appropriate because of its fast onset of action. Chang and Hudesman^[19]^ reported that vedolizumab or ustekinumab as first-line therapy was a reasonable option for a patient with a more “moderate” disease naïve to biologics. However, the addition of PSL may broaden the adaptation criteria of vedolizumab or ustekinumab for UC. Meanwhile, administration of PSL combined with ustekinumab and AZA for early remission induction is clearly more immunosuppressive and increases the risk of infection. Currently, clear criteria for the indication of PSL addition after the initiation of new biologics for UC remain unavailable. Therefore, clinicians should carefully consider the safety of PSL combinations in the future. The effects of early symptom improvement in children with anti-TNF failure should also be investigated.

In conclusion, ustekinumab was safe and effective against moderately active UC with anti-TNF-failure in the present case. Adding PSL to improve the responsiveness to UC induction therapy requires further accumulation of cases to validate its safety.

## Acknowledgments

The authors would like to thank the outpatient nurses and medical support staff at our hospital. The authors thank the patient's family for providing consent and for granting permission to draft and publish this case report.

## Author contributions

**Conceptualization:** Toshihiko Kakiuchi.

**Data curation:** Toshihiko Kakiuchi, Masato Yoshiura.

**Formal analysis:** Toshihiko Kakiuchi.

**Investigation:** Toshihiko Kakiuchi, Masato Yoshiura.

**Methodology:** Toshihiko Kakiuchi, Masato Yoshiura.

**Project administration:** Toshihiko Kakiuchi.

**Supervision:** Toshihiko Kakiuchi.

**Validation:** Toshihiko Kakiuchi.

**Writing – original draft:** Toshihiko Kakiuchi.

**Writing – review & editing:** Masato Yoshiura.
